# Supplementary material for: Phenethyl Isothiocyanate-Conjugated Chitosan Oligosaccharide Nanophotosensitizers for Photodynamic Treatment of Human Cancer Cells
Source: Int J Mol Sci. 2022 Nov 9;23(22):13802. doi: 10.3390/ijms232213802 (PMC9693342; doi:10.3390/ijms232213802)
Supplement: Supplementary file 1 [file ijms-23-13802-s001.zip › ijms-1913499-supplementary.pdf]

Supplementary Materials

Experimental

Materials

Chitosan oligosaccharide (COS), were purchased from Tokyo Chemical Industry (TCI) Co., LTD. (Tokyo, Japan). Deacetylation degree of chitosan oligosaccharide was approximately 94 % and its molecular weight was approximately 1,832 g/mol.

Characterization of chitosan

^{1}H NMR spectra and ^{13}C NMR spectra of COS and chitosan 15k (500 mHz Agilent ProPulse NMR system, Agilent Tech. Inc., Santa Clara, CA, USA) was employed to confirm chemical composition and synthesis procedures of conjugates. Each component and conjugates were dissolved in mixtures of D_2O/DMSO (1/1, v/v) for analysis.

Quantitative NMR spectra was also employed to analyze molecular weight of COS using dimethylmalonic acid in D_2O/DMSO (1/1, v/v).

Matrix-Assisted Laser Desorption Ionization Mass (MALDI TOF/TOF MS, ultraflextreme Redefine MALDI-TOF/TOF performance, Bruker Co., Billerica, MA, USA) spectrometer system was employed to characterize of COS.

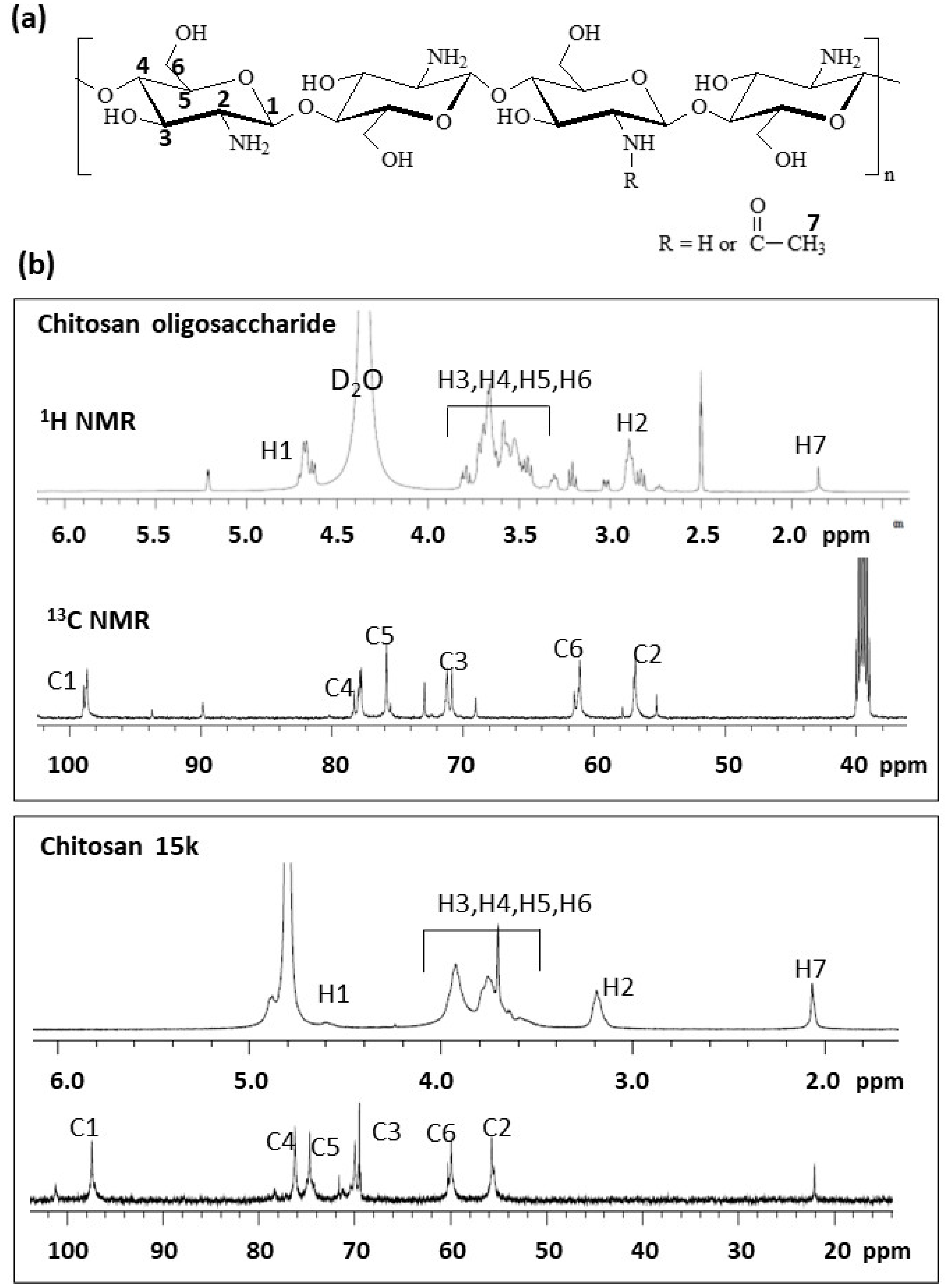

Figure S1. Chemical structure (a) and ^{1}H or ^{13}C NMR spectra of COS and chitosan 15k. (b).

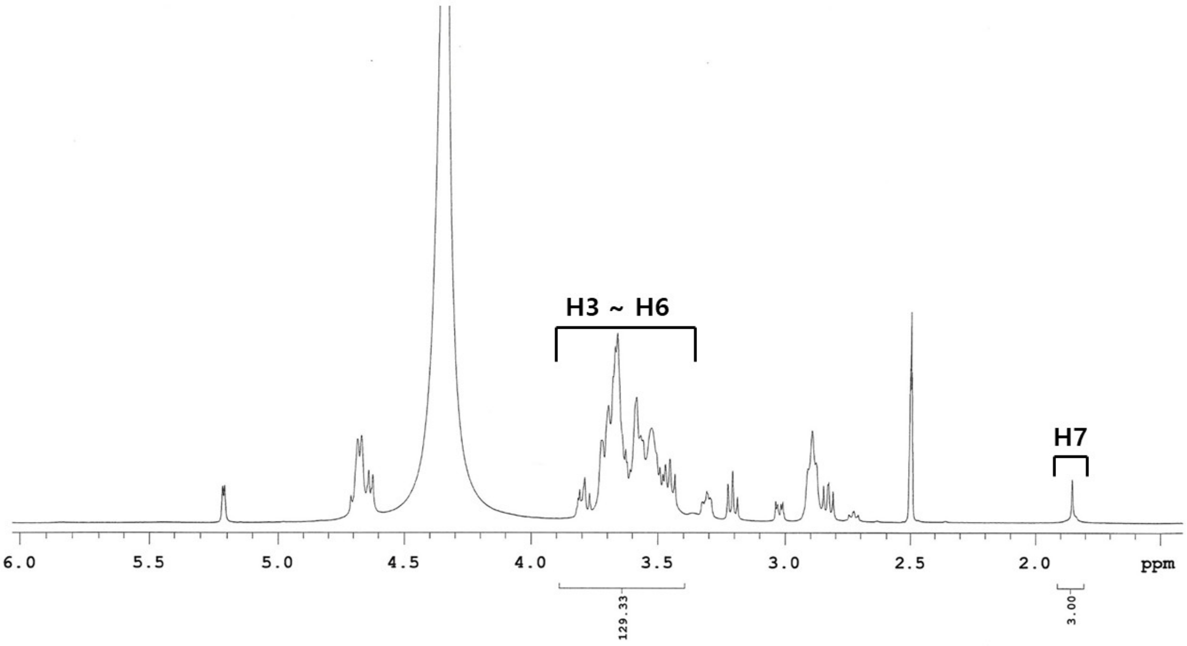

Figure S2. ^{1}H NMR spectra of COS for evaluation of deacetylation degree of COS. To calculate deacetylation degree of COS, integral value of (H3 ~ H6) and H7 was compared and then value of deacetylation degree was calculated as 96.13.

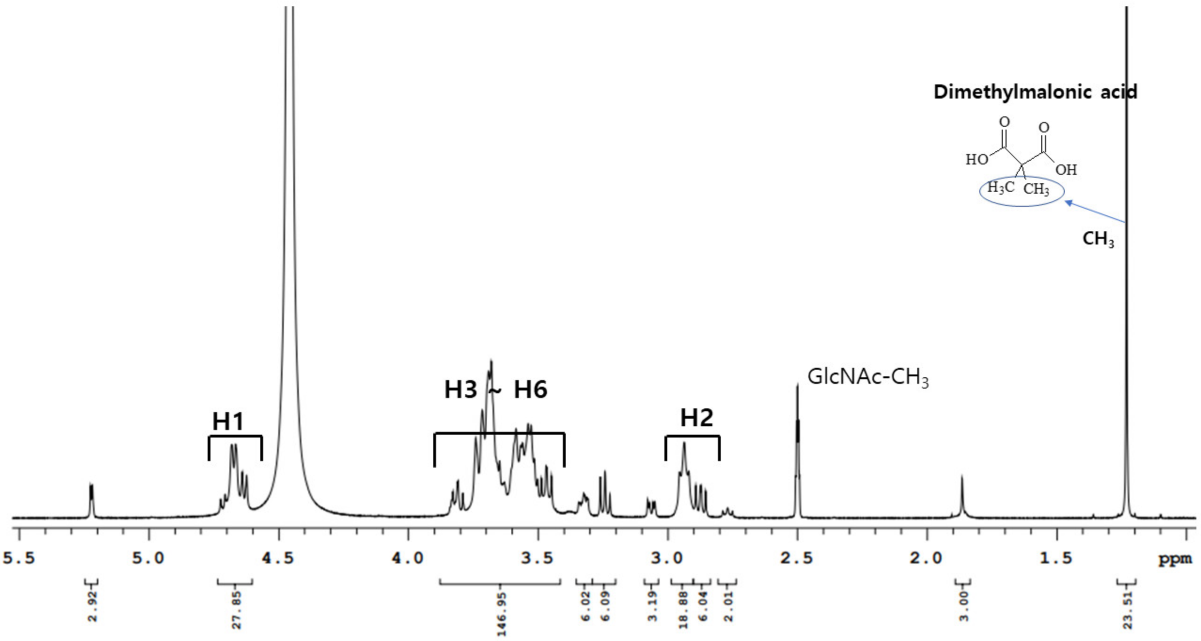

Figure S3. qNMR spectra of COS for evaluation of MW. For evaluation of COS M.W., dimethylmalonic acid was used as a standard material. Th specific peaks of dimethylmalonic acid was observed in 1.23 ppm. Based on integral value in NMR and M.W. of dimethylmalonic acid, MW of COS was calculated and then M.W. to 1,832 g/mol. COS and dimethylmalonic acid were dissolved in 1 ml DMSO/D_2O mixtures (1/1, v/v). M.W. of COS was calculated with following equation:

$$C_x = \frac{I_x}{I_{cal}} \times \frac{N_{cal}}{N_x} \times C_{cal}$$

I : integral area

N : number of nuclei

C : concentration of the compound of interest (x), calibrant (cal)

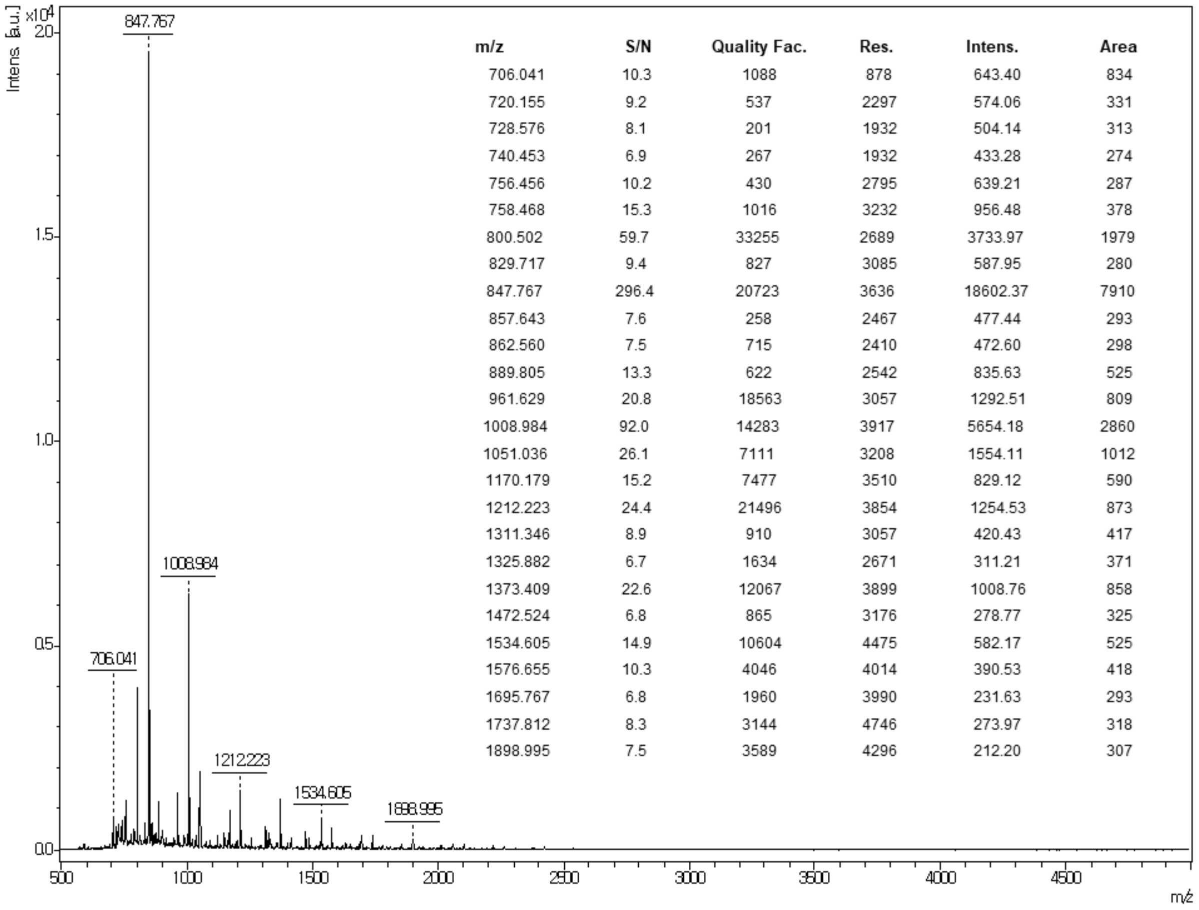

m/z	S/N	Quality Fac.	Res.	Intens.	Area
706.041	10.3	1088	878	643.40	834
720.155	9.2	537	2297	574.06	331
728.576	8.1	201	1932	504.14	313
740.453	6.9	267	1932	433.28	274
756.456	10.2	430	2795	639.21	287
758.468	15.3	1016	3232	956.48	378
800.502	59.7	33255	2689	3733.97	1979
829.717	9.4	827	3085	587.95	280
847.767	296.4	20723	3636	18602.37	7910
857.643	7.6	258	2467	477.44	293
862.560	7.5	715	2410	472.60	298
889.805	13.3	622	2542	835.63	525
961.629	20.8	18563	3057	1292.51	809
1008.984	92.0	14283	3917	5654.18	2860
1051.036	26.1	7111	3208	1554.11	1012
1170.179	15.2	7477	3510	829.12	590
1212.223	24.4	21496	3854	1254.53	873
1311.346	8.9	910	3057	420.43	417
1325.882	6.7	1634	2671	311.21	371
1373.409	22.6	12067	3899	1008.76	858
1472.524	6.8	865	3176	278.77	325
1534.605	14.9	10604	4475	582.17	525
1576.655	10.3	4046	4014	390.53	418
1695.767	6.8	1960	3990	231.63	293
1737.812	8.3	3144	4746	273.97	318
1898.995	7.5	3589	4296	212.20	307

Figure S4. MALDI TOF/TOF MS spectrum of COS.
